# Recombinant Pseudorabies Virus with TK/gE Gene Deletion and Flt3L Co-Expression Enhances the Innate and Adaptive Immune Response via Activating Dendritic Cells

**DOI:** 10.3390/v13040691

**Published:** 2021-04-16

**Authors:** Lun Yao, Qiao Hu, Siqi Chen, Tong Zhou, Xuexiang Yu, Hailong Ma, Ahmed. H. Ghonaim, Hao Wu, Qi Sun, Shengxian Fan, Qigai He

**Affiliations:** 1 State Key Laboratory of Agricultural Microbiology College of Veterinary Medicine, Huazhong Agricultural University, Wuhan 430070, China; yaolun@webmail.hzau.edu.cn (L.Y.); 2016302110181@webmail.hzau.edu.cn (Q.H.); chensiqi@webmail.hzau.edu.cn (S.C.); zhoutong61@webmail.hzau.edu.cn (T.Z.); yuxuexiang@webmail.hzau.edu.cn (X.Y.); littlelate@webmail.hzau.edu.cn (H.M.); drcahmed91@gmail.com (A.H.G.); vet_wuhao@webmail.hzau.edu.cn (H.W.); sunqi1019@webmail.hzau.edu.cn (Q.S.); fanshengxian@mail.hzau.edu.cn (S.F.); 2 The Cooperative Innovation Center for Sustainable Pig Production, Huazhong Agricultural University, Wuhan 430000, China; 3 Desert Research Center, Cairo 11435, Egypt

**Keywords:** emerging PRV, dendritic cells, vaccine, immune response

## Abstract

Owing to viral evolution and recombination, emerging pseudorabies virus (PRV) strains have caused unprecedented outbreaks in swine farms even when the pigs were previously vaccinated, which might indicate that traditional vaccines were unable to provide effective protection. The development of safe and efficacious vaccines presents prospects to minimize the clinical signs and eventually eradicate the infection. In this study, we used an emerging PRV strain, HNX, as the parental strain to construct a recombinant PRV with TK/gE gene deletion and Fms-related tyrosine kinase 3 ligand (Flt3L) expression, named HNX-TK^−^/gE^−^-Flt3L. HNX-TK^−^/gE^−^-Flt3L enhanced the maturation of bone marrow derived dendritic cells (DCs) in vitro. Significantly more activated DCs were detected in HNX-TK^−^/gE^−^-Flt3L-immunized mice compared with those immunized with HNX-TK^−^/gE^−^. Subsequently, a remarkable increase of neutralizing antibodies, gB-specific IgG antibodies, and interferon-gamma (IFN-γ) was observed in mice vaccinated with HNX-TK^−^/gE^−^-Flt3L. In addition, a lower mortality and less histopathological damage were observed in HNX-TK^−^/gE^−^-Flt3L vaccinated mice with upon PRV lethal challenge infection. Taken together, our results revealed the potential of Flt3L as an ideal adjuvant that can activate DCs and enhance protective immune responses and support the further evaluation of HNX-TK^−^/gE^−^-Flt3L as a promising PRV vaccine candidate.

## 1. Introduction

Pseudorabies virus (PRV) is an alpha-herpesvirus that causes neurological and respiratory system disorders in young piglets and abortion in pregnant sows, which leads to severe economic losses in the swine industry [1,2]. The PRV genome is double-stranded DNA of approximately 145 kb in size, which contains almost 70 open reading frames (ORFs) that encode 70–100 viral proteins, including structural, non-structural, and virulence-associated proteins [3,4]. 

Vaccination is a global tool to control pseudorabies (PR); however, as a result of PRV evolution and recombination, emerging PRV strains have caused large-scale outbreaks of PR in China since 2011 [5,6,7,8]. For example, in Shandong province, one of the most vital pig-breeding regions in China, 52.7% and 91.5% of the serum samples were positive for PRV-gE and –gB from 2015 to 2018 [9], while in Heilongjiang province, 16.3% and 84.5% were positive from 2013 to 2018 [10]. In Europe, around 40% of the wild boar in Spain were infected [11], and 40 out of 54 sera (74%) scored positive for anti-gB PRV antibodies from the 2018 to 2019 hunting seasons (from November to January) in Tuscany, Italy [12]. Recently, disease in swine-farms workers were associated with emerging PRV strains; thus, the prevention of PR in swine may decrease the risk of human infection [13,14,15]. PRV Bartha K61, a vaccine strain, appears to not provide rapid and complete protection from emerging strains of PRV, and thus one of the new vaccine development strategies is to improve the immunogenicity of a vaccine by using a PRV emerging strain as the parental strain to elicit a stronger immune response. 

Dendritic cells (DCs) are considered the most potent antigen-presenting cells and a key element of both the innate and adaptive immunity to viral infections [16]. DCs reside in and travel across tissues, and, once activated, antigen-loaded DCs migrate to secondary lymphoid tissues, where they can where they can activate antigen-specific T cell responses. The cytokine Fms-related tyrosine kinase 3 ligand (Flt3L) is a hematopoietic growth factor with well-established functions in hematopoietic cell development, such as driving DC commitment and promoting lymphoid lineage development [17]. A previous study indicated that mice deficient in Flt3L showed about 4–14-fold lower numbers of DCs in lymphoid organs and 5-fold lower quantities of natural killer cells. Exogenous Flt3L treatment increased the numbers of early myeloid and lymphoid progenitors and also increased the quantities of peripheral DCs [18]. There is convincing evidence to demonstrate that Flt3L and its receptor (Flt3 and fetal liver kinase-2) are important regulators of homeostatic DCs division in the periphery [17,19]. Research reported that vaccine-induced protective immunity is controlled by a Flt3L-dependent pathway that can mediate expansion of DCs and enhance T cell activation [20,21,22]. To exploit the ability of Flt3L to expand DCs, we constructed a recombinant PRV with TK/gE gene deletion and Flt3L co-expression. Our study characterized both the role of DC activation in PRV vaccination and potential of Flt3L as an adjuvant in the PRV vaccine-induced immune response. 

Recently, clustered regularly interspaced short palindromic repeats (CRISPR) and CRISPR-associated protein 9 (Cas9) have been applied to virus gene modification [23,24,25]. Following guide RNA (gRNA) recognition of a 20 nucleotide target sequence, Cas9 and its mutant Cas9 nickase (Cas9n) protein break or nick the targeted DNA [26,27,28]. Subsequently, this broken site can be repaired through the homology-directed repair (HDR) pathway and the indel target region from a homologous DNA donor [23,26,29]. The Cre-Lox system is another site-specific modified technology, where the cre enzyme derived from a bacteriophage can recognize a specific lox site and remove specific DNA between Lox sites [30]. The conjunctive use of the CRISPR/Cas9 and Cre/Lox systems accelerated our vaccine development.

## 2. Materials and Methods 

### 2.1. Cells and Virus

PK-15 cell and HEK293T cell were originally purchased from the Cell Bank of the Chinese Academy of Science (Shanghai, China and were maintained in Dulbecco’s modified Eagle’s medium (DMEM, Gibco, Thermo Fisher Scientific, Waltham, MA, USA) supplemented with 10% fetal bovine serum (FBS, Natocor, Cordoba, Argentina) at 37 °C in a humidified incubator containing 5% CO_2_. The HNX strain (GenBank accession no. KM189912.1) was isolated from a pig farm in the Henan province of China [31]. The TK and gE gene deletion mutant of the HNX strain, named HNX-TK^−^/gE^−^, was kindly provided by Prof. Gang Cao, Huazhong Agricultural University [25]. 

### 2.2. Plasmid Construction

Single guide RNA (sgRNA) of the TK and gE sequences were designed using the CRISPR design tool (http://crispr.mit.edu/, Feng Zhang’s Lab, access date: 10 December 2018) and were cloned into a sgRNA/Cas9 expression vector (Addgene plasmid px459) by using BbsI restriction enzyme sites. The HNX genome was used as a PCR template for amplifying of homologous arms. The Flt3L gene was amplified from primary alveolar macrophage (PAM) cells complementary (c) DNA by PCR. The donor template (gEhm1-loxP-GFP-loxP-gEhm2 and TKhm1-Flt3L-loxN-CMV promoter-mCherry-loxN-TKhm2) were constructed by overlapping PCR. Cre recombinase plasmid (Addgene plasmid pCS-Cre) and the control plasmids (Addgene plasmid pCS) was provided by Prof. Gang Cao, Huazhong Agricultural University. All the sequences of the primers and sgRNAs are listed in Table S1, and the sequences of gE and TK gene recombination donor templates are listed in Figures S1 and S2.

### 2.3. CRISPR/Cas9-Mediated Gene Insertion

HEK293T cells were cultured in 6-well plates grown to approximately 50% confluence, and then transfected with gRNA-TK (0.5 µg), gRNA-gE (0.5 µg), gEhm1-loxP-eGFP-loxP-gEhm2 (1.0 µg), and TKhm1-Flt3L-loxN-CMV promoter-mCherry-loxN-TKhm2 (1.0 µg) per well. The Lipofectamine2000 transfection reagent (Invitrogen, Shanghai, China) was used for transfection of plasmids, following the manufacture’s protocol. After transfection, the HEK293T cells were infected with HNX at 0.01 multiplicity of infection (MOI) to acquire recombinant HNX-TK^−^/gE^−^-Flt3L with eGFP and mCherry.

### 2.4. Fluorescence-Activated Cell Sorting (FACS)

The FACS was performed on a MoFlo XDP sorter (Beckman Coulter, Inc., Brea, CA, USA) following the manufacturer’s protocol. PK-15 cells were infected with the first generation recombinant PRV at 0.01 MOI. After 36 h, the single cells expressing fluorescent proteins were sorted into a 96-well plate pre-seeded with 5000 PK-15 cells.

### 2.5. Virus Preparation, Propagation and Plaque Purification

Virus-infected PK-15 cells were maintained in DMEM with 2% FBS for 24 h. After three rounds of freeze–thaw, the cell lysate was centrifuged for 10 min at 5000× *g*. The supernatant was used for next propagation or stored at −80 °C. For virus purification, the PK-15 cells were grown to approximately 80% confluence, then infected with recombinant virus, and covered with mixture of 2 × DMEM (Gibco, Thermo Fisher Scientific), 2% low melting-point agarose (HyClone, GE Healthcare Life Sciences, Inc., Stockholm, Sweden), 1% FBS (Natocor), 100 units/mL penicillin (Genom, Hangzhou, China), and 100 µg/mL streptomycin (Genom). The plaques were observed via fluorescence microscopy. After 36–48 h, well-separated plaques were picked up by a 10 µL pipette tip and transferred into micro-tubes containing 300 μL serum free DMEM for the next propagation or plaque purification.

### 2.6. Cre/Lox-Mediated Marker Genes Excision

HEK293T cells were seeded into 6-well plates and grown to approximately 50% confluence, then transfected with 2.5 ng of pCS-Cre or pCS plasmids with Lipofectamine 2000. After 24 h, the transfected cells were infected with recombinant PRV at 0.01 MOI. Virus-infected HEK293T cells were maintained in DMEM with 2% FBS for 36 h to remove eGFP and mCherry for creating HNX-TK^−^/gE^−^-Flt3L.

### 2.7. RNA Isolation and Quantitative Real-Time PCR (RT-qPCR)

For the detection of mRNA of Flt3L, total RNAs were extracted from infected PK-15 cells using TRIzol (Takara, Dalian, China) following the manufacturer’s protocol. Total RNAs were reverse-transcribed using the Two-Step MMLV RT-PCR kit from GeneMark (Shanghai, China), and RT-qPCR was performed to determine transcription of Flt3L with Roche Lightcycler 96 using SYBR Green real-time PCR master mix from GeneMark (Shanghai, China) according to the manufacturer’s instructions. The specific primers for Flt3L amplification were: 5′CTGCTGCTGCTGCTGCTGAG-3′ and 5′GAGATGTTGGCCTGGACGAAGC-3′, the primers for GAPDH were: 5′CTTCCGTGTCCCTACTGCCAAC-3′, and 5′- GACGCCTGCTTCACCACCTTCT-3′.

### 2.8. Western Blot Analysis

HNX-TK^−^/gE^−^-Flt3L- or HNX-infected PK-15 cells were maintained in DMEM with 2% FBS for 24 h. Then, PK-15 cells were lysed in 1 mL of ice-cold tissue lysis buffer (Tris-buffered saline, 1.5% Triton X-100, 0.5% deoxycholic acid, 0.1% sodium dodecyl sulfate, protease inhibitor cocktail, and 1 mM phenylmethanesulfonyl fluoride). After centrifugation (12,000× *g*, 20 min, 4 °C), the supernatants were collected and the protein concentrations were determined. The protein samples were loaded onto 10% SDS-polyacrylamide gel and transferred onto nitrocellulose membranes. The blots were blocked by skim milk (BD, Bioscience, Inc., Saint Louis, MO, USA), and this was followed by incubation with rabbit anti-Flt3L polyclonal antibody (Bioss, Beijing, China), mouse anti-gD monoclonal antibody (Keqian Ltd., Wuhan, China), and mouse anti-β-actin monoclonal antibody (Bioss).

### 2.9. Immunofluorescence Assay (IFA)

PK-15 cells were cultured in 96-well plates and maintained in DMEM containing 2% FBS. The confluent cell monolayers were respectively infected with HNX-TK^−^/gE^−^-Flt3L or HNX-TK^−^/gE^−^ at 0.05 MOI. After 24 h, PK-15 cells were fixed with ethanol for 30 min at −20 °C, followed by incubation with rabbit anti-Flt3L polyclonal antibody (Bioss) or mouse anti-gB monoclonal antibody (Keqian) for 1 h at 37 °C. After three washes with PBS (Gibco, Thermo Fisher Scientific), the cells were respectively stained with Cy3-conjugated goat anti-rabbit IgG (Abclonal, Wuhan, China) or FITC-conjugated goat anti-mouse IgG (Abclonal) for 30 min at 37 °C. Images were captured using an Olympus CK40 microscope.

### 2.10. Replication Kinetics of the Rescued Recombinant PRV

PK-15 cells were cultured in 24-wells plate and infected with HNX-TK^−^/gE^−^-Flt3L and HNX at 0.1 MOI. The supernatant and cells were harvested at 4, 8, 12, 16, 20, 24, 28, 32, 36, 40, 44 and 48 h post-infection. The virus titers of HNX-TK^−^/gE^−^-Flt3L were determined according to the Reed–Muench method to produce gaining a growth curve. Three independent experiments were conducted. 

### 2.11. Collection of Bone Marrow-Derived DCs

Mouse bone marrow-derived DCs were collected as described before [32,33]. Briefly, BALB/c mice were euthanized and the femur was separated. The bones were left in 75% ethanol for 5 mins for disinfection and washed with PBS. Then, both ends were cut with scissors, and the marrow was flushed with PBS using a 10 mL syringe with a 0.45 mm diameter needle. Bone marrow, harvested from the femur, was dissociated into single-cell suspensions by vigorous pipetting. Cells were counted on a hemocytometer and cultured in RPMI 1640 medium (Gibco, Thermo Fisher Scientific, Inc., Waltham, MA, USA) supplemented with 10% FBS, 20 ng/mL recombinant mouse GM-CSF (Peprotech, Rocky Hill, CT, USA) and 10 ng/mL recombinant mouse IL-4 (Peprotech) in 6-well plates at a density of 2 × 10^5^ per well. At 2, 4, and 6 days post cultivation, fresh DC medium was replenished. The cells were collected and re-cultured in 12 well plates at a density of 10^6^ per well at 7 days post cultivation. After 24 h, three well of DCs were stained with FITC anti-mouse CD11c antibodies (BioLegend, San Diego, CA, USA) for flow cytometry analysis. The purity check, though identification by the percent of CD11c positive cells, was over 85% (data not shown). The DCs were appropriate for use in the further studies. 

### 2.12. Sample Preparation and Flow Cytometry Analysis

To assess the activation of bone marrow-derived DCs, DCs were cultured in 12-well plates and infected with HNX-TK^−^/gE^−^-Flt3L or HNX-TK^−^/gE^−^ at 0.01 MOI. Lipopolysaccharide (LPS) was used as a positive control. After 24 h, DCs were harvested for flow cytometry analysis. To examine the activation of DCs in vivo, three-week-old female BALB/c mice were divided into three groups (ten mice each). Groups of BALB/c mice (*n* = 10) were stimulated with 1 × 10^5^ TCID_50_ of HNX- TK^−^/gE^−^-Flt3L, HNX- TK^−^/gE^−^, or DMEM medium. Inguinal lymph nodes were collected from the mice at 36 and 72 h post infection (hpi). 5 mice were euthanized and inguinal lymph nodes were separated at each timepoint. 

The collected lymph nodes were homogenized and filtered through a 40 µm nylon filter, then washed with PBS. Single-cell suspensions of lymph nodes cells and bone marrow-derived DCs were prepared as 10^6^ cells/mL with 0.2% BSA in PBS and stained with anti-mouse CD86-PE, CD11c-FITC, MHC class II-PE/Cy7, CD80-APC monoclonal antibodies (all from BioLegend, San Diego, CA, USA) at 4 °C for 30 min. After washing with PBS, the cells were fixed in 4% paraformaldehyde for 30 min. Flow cytometry was performed on an LSR-II flow cytometer (BD, Bioscience, Inc.) and analyzed using FlowJo software [34].

### 2.13. Mice Immunization and Challenge Experiment

Six-week-old female BALB/c mice were divided into three groups (12 mice/each), The caudal thigh muscles of BALB/c mice were intramuscularly injected with 1 × 10^5^ TCID_50_ of HNX-TK^−^/gE^−^-Flt3L, HNX-TK^−^/gE^−^, or DMEM in 100 μL. After two weeks of the first immunization, a second immunization was carried out. At 21 days post booster immunization, the mice were challenged with 1 × 10^5^ TCID_50_ of HNX strain in a volume of 50 μL by footpad injection. At 72 h post PRV challenge, 4 out of 12 mice were randomly selected in each group for histopathology analysis, and the other mice were observed for 2 weeks.

### 2.14. Antibody Detectionand Cytokine Detection

In the mouse immunization and challenge experiment, blood samples were collected from mice by submandibular bleeding at 0, 7, 14, 21, 28, 35 and 38 days post first immunization. For plasma collection, approximately 100 µL of blood was transferred into 1.5-mL EDTA capillary collection tubes (BD, Bioscience, Inc., Franklin Lakes, NJ, USA). The samples were first centrifuged for 10 minutes at 1500× *g* and 4 °C to separate the cells from the plasma and then for 15 min at 2000× *g* and 4 °C to deplete the platelets. The plasma samples were stored at −80 °C. For serum collection, approximately 300 µL of blood was transferred into 1.5-mL tubes (Fengqin, Guangzhou, China) and maintained at 4 °C for 24 h. Then, the samples were centrifuged for 10 minutes at 2000× g and 4 °C, and the supernatant was collected and stored at −80 °C for further study. 

The PRV-specific gB antibodies were evaluated using commercial blocking ELISA kits according to the manufacturer’s protocol (IDEXX, Westbrook, MA, USA). Same ELISA kits were used to analyze the isotypes of IgG (IgG1 and IgG2a) in the serum. Conjugated anti-gB antibody was replaced by HRP-conjugated goat anti-mouse IgG1 or IgG2a (ABclonal) at a dilution of 1:1000. The results were read at 450 nm. The IFN-γ, IL-6, MCP-1, and CXCL10 levels in the plasma were evaluated using commercial ELISA kits from Cusabio (Wuhan, China), and the analysis was performed according to the manufacturer’s protocol.

### 2.15. Neutralizing Antibody Assay

The presence of neutralizing antibodies against PRV were tested as described previously [35]. Briefly, the serum was inactivated at 56 °C for 30 mins, and 50 μL of serially diluted serum was mixed with an equal volume of the HNX strain containing 100 TCID_50_. The mixture was incubated at 37 °C with 5% CO_2_ for 1 h, and then transferred to PK15 cells to incubate for further 3–5 days. Cells were microscopically examined to determine the cytopathic effect (CPE). Neutralizing antibody titers were expressed as the highest dilution that reduced the CPE of HNX strain by 50% compared with non-neutralized controls, and calculated as the average of three measurements according to the Reed–Muench method.

### 2.16. Histopathology

In the mouse immunization and challenge experiment, 4 out of 12 mice were randomly selected in each group for histopathology analysis. BALB/c mice were euthanized, and brain tissues were collected and fixed with 4% paraformaldehyde solution at room temperature for 2 days. The brain tissues were transferred to 70% ethanol, placed in processing cassettes, dehydrated through a serial alcohol gradient, and embedded in paraffin wax blocks. 5 μm thick sagittal brain sections were cut and every tenth section in the series spanning from Bregma lateral 0.5 mm to 1.49 mm were stained with a Hematoxylin-Eosin (HE) Staining Kit (Solarbio, Beijing, China) according to the instructions. The average numbers lymphocytic infiltration foci, glial nodules and hemorrhage foci in per section were quantified. Quantification of neuronophagia in cerebral cortex was performed with an ocular grid in 400× magnification and the percentage of neurons surrounded by neuronophagia lymphocytes or glial cells was calculated. The thickness of leptomeninges was determined in five random fields at per section and quantitative analysis was performed with software Image-Pro Plus 6.0 (Media Cybernetics, lnc., Rockville, MD, USA).

### 2.17. Statistical Analysis

The significant differences of the data were analyzed using two-way ANOVA and/or a t-test in the GraphPad Prism (version 5.00) software (San Diego, CA, USA). Significance was defined as *p*-values less than 0.05, and *p*-values less than 0.01 were regarded as highly significant.

## 3. Results

### 3.1. Construction of a Recombinant PRV by CRISPR/Cas9 and Cre/Lox System

**Figure 1:**
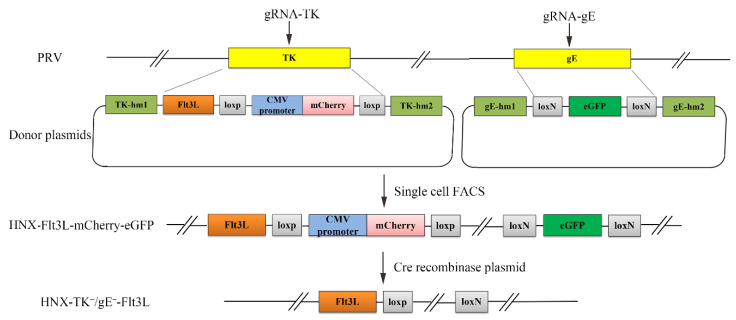
Schematic diagram of the recombinant pseudorabies virus (PRV) development strategy. The PRV virulence-related genes TK and gE were simultaneously replaced via the CRISPR/Cas9 system by using DNA templates that included fluorescent marker genes (mCherry and eGFP) and the Flt3L gene. Then, single cell fluorescence-activated cell sorting (FACS) was used to accelerate the screening and purification of the recombinant PRV virus. Subsequently, both fluorescent marker genes were excised using the Cre/Lox system.

To simultaneously carry out both foreign gene knock-in and virulence-related gene deletion in a highly efficient method, two gene edit systems, CRISPR/Cas9 and Cre/Lox were applied. The single cell FACS technique was also performed to accelerate the purification of recombinant PRV. As the most important virulence-related genes, the gE and TK genes are usually deleted to ensure the safety of attenuated PRV vaccines [25,36]. Foreign gene expression was driven by an endogenous viral promoter with a strategy that our previous study demonstrated to have genetic stability (date not shown). The eGFP reporter was driven by an endogenous viral promoter, which could be observed only after precise CRISPR/Cas9-mediated homologous recombination. Under the Cre enzyme expression, LoxP and LoxN pairs were applied to facilitate mCherry and eGFP gene excision, respectively (Figure 1).

**Figure 2:**
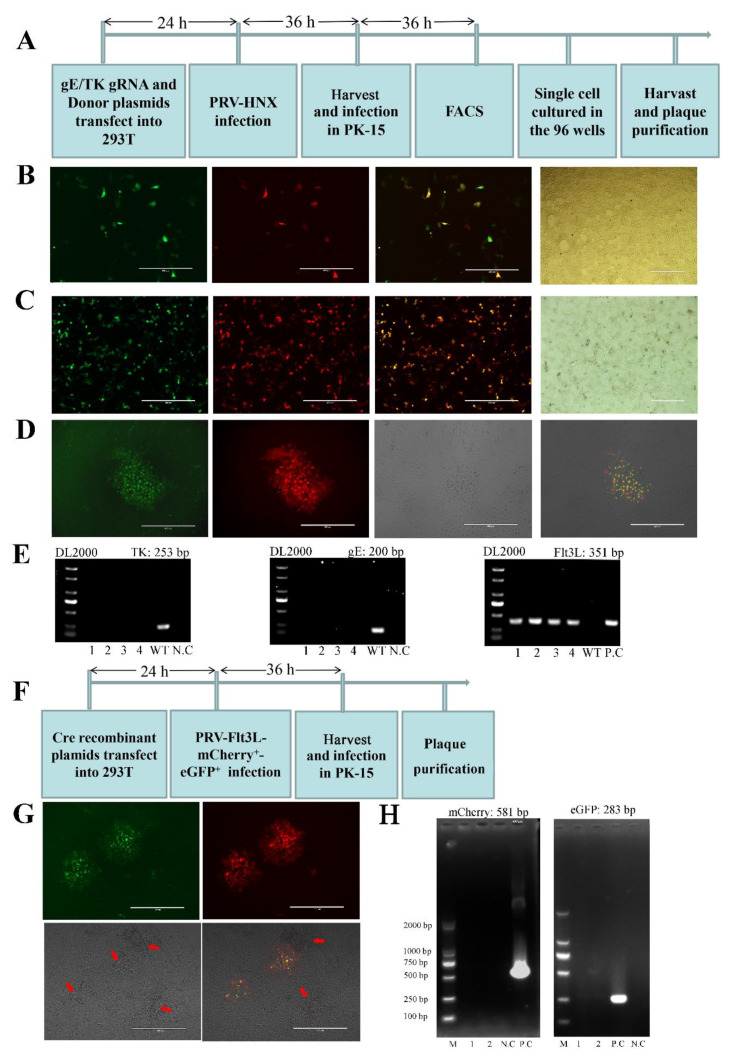
Construction of recombinant HNX-TK^−^/gE^−^-Flt3L. (**A**) Process of CRISPR/Cas9-assisted TK and gE gene recombination. (**B**) Single guide RNA (sgRNA) and donor plasmids were co-transfected into 293T cells, and, 24-h post transfection, the cells were infected with PRV HNX strain at 0.01 MOI. After 36 h, fluorescence microscopy was used to observe the fluorescent marker gene expression. (**C**) After single cell FACS, single cells were plated into 96-well plates pre-seeded with PK-15 cells, and, after 24 h, the fluorescence was observed. (**D**) Plaque purification of the recombinant PRV in agarose-DMEM cultured plates. (**E**) PCR verification of the TK, gE, and Flt3L genes. **Lanes 1**–**4—**HNX-TK^−^/gE^−^-Flt3L-mCherry^+^-eGFP^+^. N.C., negative control. P.C., Positive control. (**F**) Process of double fluorescent gene excision using the Cre/Lox system. (**G**) Plaque purification for the double fluorescent gene excision virus. Virus plaques are indicated with arrows. (**H**) PCR verification of the mCherry and eGFP genes. Lanes 1,2: HNX-TK^−^/gE^−^-Flt3L. N.C., negative control. P.C., Positive control.

To obtain recombinant PRV with TK/gE gene deletion and Flt3L co-expression, CRISPR/Cas9 and Cre-lox-based systems were applied to produce the vaccine candidate. gRNA-TK, gRNA-gE, TKhm1-Flt3L-loxN-CMV promoter-mCherry-loxN-TKhm2, and gEhm1-loxP-GFP-loxP-gEhm2 were co-transfected into HEK293T cells, and 24-h post transfection, the cells were infected with PRV HNX strain at 0.01 MOI. Then, the cells were collected and subjected to single-cell FACS technique to purify the recombinant PRV. Plaque purification was performed to obtain the pure recombinant virus (Figure 2A). 

Fluorescence detection showed that the recombinant viruses (named HNX-TK^−^/gE^−^-Flt3L-mCherry^+^-eGFP^+^) expressing red and green fluorescence were successfully visualized (Figure 2B). Then, the recombinant PRV viruses with red or green signals were harvested and used to infect PK-15 cells. The infected cells were digested into single cells and then subjected to FACS. Infected PK-15 cells co-expressing mCherry and eGFP were seeded into 96-well plates (Figure 2C). In addition, one round of plaque purification was performed to obtain the pure recombinant virus (Figure 2D). Then, four well-separated plaques were picked up using pipette tips for the next propagation and PCR assay. Positive amplification of Flt3L was observed using in the HNX-TK^−^/gE^−^-Flt3L template, while no amplifications of the TK and gE genes were found. However, TK and gE could be amplified from the parental PRV (Figure 2E). To completely remove the reporter genes from the vaccine candidate, the Cre/lox system was used to remove the eGFP and mCherry indicators. HEK293T cells were transfected with pCS-Cre or pCS plasmid. At 24 h post transfection, transfected HEK293T cells were infected with HNX-TK^−^/gE^−^-Flt3L-mCherry^+^-eGFP^+^ at 0.01 MOI. After 36 h of infection, infected PK-15 cells were harvested. Then, the fluorescence gene was excised through three rounds of plaque purification (Figure 2F). The HEK293T cells harboring pCS-Cre plasmids exhibited significant less fluorescence compared to those transfected with the pCS plasmids (Figure 2G). To verify the purity of the recombinant viruses, the purity of the recombinant virus was validated through PCR amplification. As shown in Figure 2H, the mCherry and eGFP gene amplifications were completely negative in HNX-TK^−^/gE^−^ but positive in HNX-TK^−^/gE^−^-mCherry^+^-eGFP^+^. 

### 3.2. Characterization of Recombinant HNX-TK^−^/gE^−^-Flt3L

**Figure 3:**
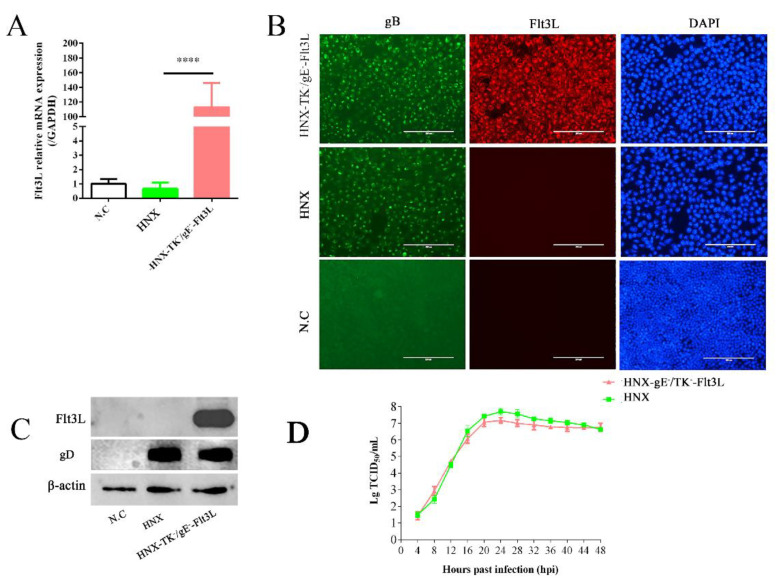
Characterization of the recombinant HNX-TK^−^/gE^−^-Flt3L. (**A**) Detection of the transcription of the Flt3L gene in HNX-TK^−^/gE^−^-Flt3L virus- and HNX-infected cells through RT-qPCR (**** < 0.001). (**B**) Detection of the Flt3L protein and viral gB protein expression through an immunofluorescence assay (IFA). Green: gB-positive cells; red: Flt3L-positive cells; blue: DAPI-stained PK-15 cell nucleus. DAPI, 4’,6-diamidino-2-phenylindole. (**C**) Western blot analysis of β-actin, gD, and Flt3L of the PK-15 cells infected with HNX-TK^−^/gE^−^-Flt3L, HNX, or DMEM as a control. (**D**) One step growth curves of HNX-TK^−^/gE^−^-Flt3L and HNX in PK-15 cells.

We examined the Flt3L expression in PK-15 cells that were infected with HNX-TK^−^/gE^−^-Flt3L. PRV HNX strain was used as the control. The Flt3L mRNA was determined using RT-qPCR. Compared with PRV HNX strain, HNX-TK^−^/gE^−^-Flt3L infection significantly increased the Flt3L mRNA transcription in PK-15 cells (Figure 3A). The presence of gB glycoprotein was detected in both HNX-TK^−^/gE^−^-Flt3L-and HNX-infected cells with IFA (Figure 3B). The expression of Flt3L protein was further determined through an IFA. After infection with HNX-TK^−^/gE^−^-Flt3L, the PK-15 cells displayed positive immunofluorescence for the Flt3L protein, whereas the cells infected with the HNX strain were negative (Figure 3B). For further assessment of the Flt3L expression, PK-15 cell lysates from HNX-TK^−^/gE^−^-Flt3L and the parental virus were analyzed using western blotting. The expression of gD glycoprotein was detected in both HNX-TK^−^/gE^−^-Flt3L-and HNX-infected cells with western blot. A band specifically recognized by the rabbit anti-Flt3L polyclonal antibody appeared in the PK-15 cells infected with HNX-TK^−^/gE^−^-Flt3L but not in those infected with HNX strain (Figure 3C). The in vitro growth kinetics of HNX-TK^−^/gE^−^-Flt3L were similar to those of HNX strain in PK-15 cells (Figure 3D).

### 3.3. Activation of Bone Marrow-Derived DCs by HNX-TK^−^/gE^−^-Flt3L In Vitro

To investigate whether HNX-TK^−^/gE^−^-Flt3L can activate DCs more strongly compared with HNX-TK^−^/gE^−^ in vitro, DCs were differentiated from murine bone marrow progenitor cells, and the purity checked by FACS was over 85% (data not shown). The DCs were cultured in 12-well plates, then infected with HNX-TK^−^/gE^−^-Flt3L and HNX-TK^−^/gE^−^ at 0.01 MOI. LPS was used as a positive control. It is well-known that CD11c is the marker of DCs. CD80, CD86, and MHC II are specific markers of activated DCs [34]. 

**Figure 4:**
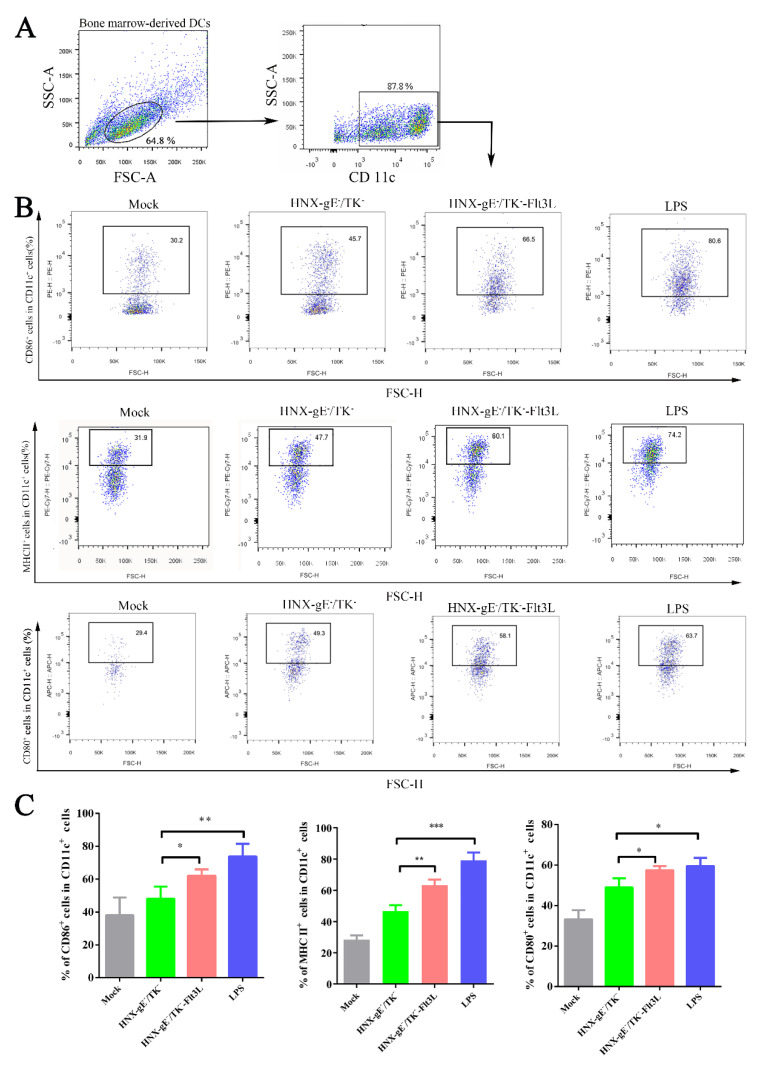
Activation of bone marrow-derived DCs by HNX-TK^−^/gE^−^-Flt3L in vitro. Bone marrow cells were harvested from BALB/c mice and maintained with GM-CSF and IL-4. LPS was used as a positive control, and the medium from untreated cells (mock) was used for negative controls. (**A**) Representative gating strategy for derived dendritic cells (DCs). (**B**) Representative flow cytometric plots of CD11c^+^- and CD86^+^-activated DCs, CD11c^+^- and MHCII^+^-activated DCs, and CD11c^+^- and CD80^+^-activated DCs. (**C**) Statistical results of DCs are summarized in the chart. The data are the means from 3.4. Activation of DCs in HNX-TK^−^/gE^−^-Flt3L immunized mice three independent experiments (* *p* < 0.05; ** *p* < 0.01; and *** *p* < 0.005).

In our representative gating strategy, CD11c^+^-positive cells were gated first, and then the activated DC markers (CD80, CD86, and MHC II) were detected using flow cytometry. The gating strategy and representative flow cytometric plots for activated DCs, such as CD11c^+^ CD86^+^, CD11c^+^ CD80^+^ and CD11c^+^ MHC II^+^ are shown in Figure 4A,B. As expected, CD80, CD86, and MHC II were significantly higher in DC cells incubated with HNX-TK^−^/gE^−^-Flt3L compared with those incubated with HNX-TK^−^/gE^−^ (Figure 4C). Overall, HNX-TK^−^/gE^−^-Flt3L significantly activated more DCs compared with HNX-TK^−^/gE^−^ in vitro.

**Figure 5:**
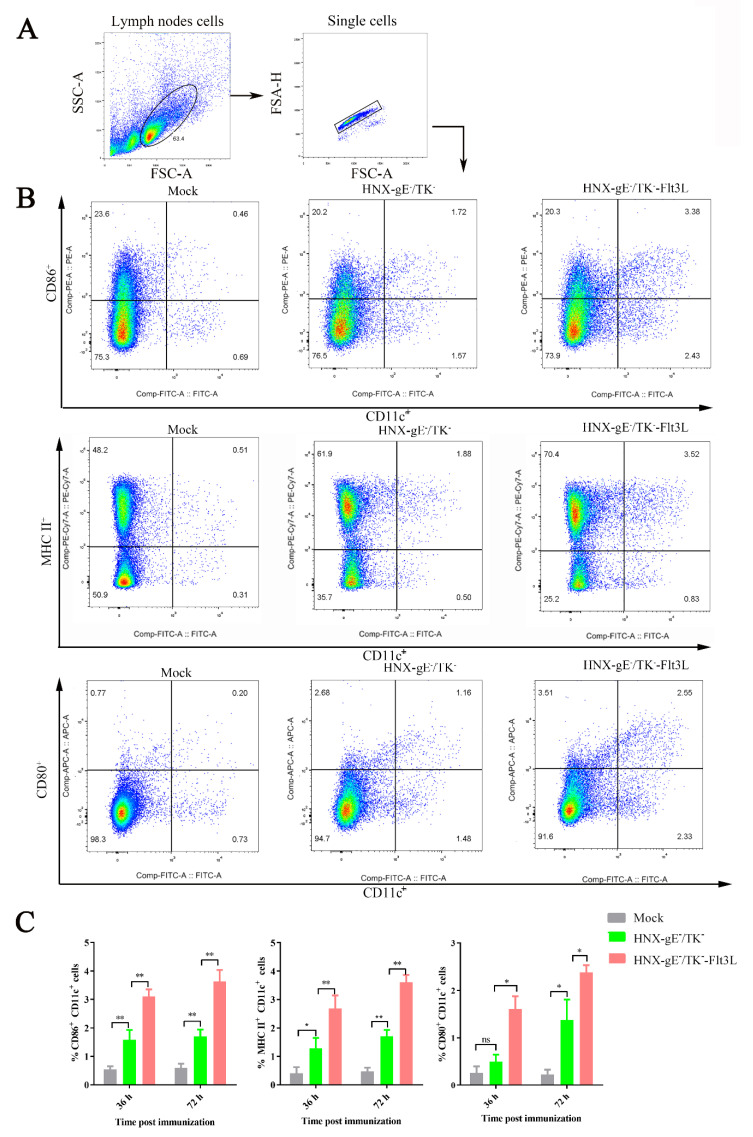
DC activation in mice immunized with HNX-TK^−^/gE^−^-Flt3L. BALB/c mice were immunized with 1 × 10^5^ TCID_50_ of HNX-TK^−^/gE^−^-Flt3L, HNX, or DMEM. The inguinal lymph nodes were collected at 36 and 72 hpi. Single cell suspensions prepared from the inguinal lymph nodes were analyzed for the presence of DCs. (**A**) Representative gating strategy for DCs. (**B**) Representative flow cytometric plots of activated DCs (CD11c+ CD86+ cells, CD11c+ MHCII+ cells, and CD11c+ CD80+ cells). (**C**) Percentages of activated DCs (CD11c^+^ CD86^+^ cells, CD11c^+^ MHCII^+^ cells, and CD11c^+^ CD80^+^ cells) are summarized statistically in the chart. The data are the means from three independent experiments (* *p* < 0.05; ** *p* < 0.01).

To examine whether HNX-TK^−^/gE^−^-Flt3L can activate more DCs in vivo, three groups of BALB/c mice (*n* = 10) were stimulated with HNX-TK^−^/gE^−^-Flt3L, HNX-TK^−^/gE^−^, or DMEM medium, respectively. Inguinal lymph nodes were collected at 36 and 72 hpi, and flow cytometry was applied to examine the fluorescence signals of the DCs marker (CD11c) and activated DCs markers (CD80^+^ and CD86^+^ or MHC II^+^). The gating strategy and representative flow cytometric plots for activated DCs (CD11c^+^ CD86^+^, CD11c^+^ CD80^+^, and CD11c^+^ MHC II^+^) at 72 hpi are shown in Figure 5A,B. Significantly more active DCs were observed at 36 and 72 hpi in the inguinal lymph nodes of mice vaccinated with HNX-TK^−^/gE^−^-Flt3L. The representative flow cytometric plots for the activated DCs (CD11c^+^ CD86^+^, CD11c^+^ CD80^+^, and CD11c^+^ MHC II^+^) at 36 and 72 hpi are summarized in Figure 5C. The above data suggest that HNX-TK^−^/gE^−^-Flt3L recruited more activated DCs in the inguinal lymph nodes of the mice vaccinated with HNX-TK^−^/gE^−^-Flt3L.

### 3.4. Immune Responses after Immunization with HNX-TK^−^/gE^−^-Flt3L in Mice

**Figure 6:**
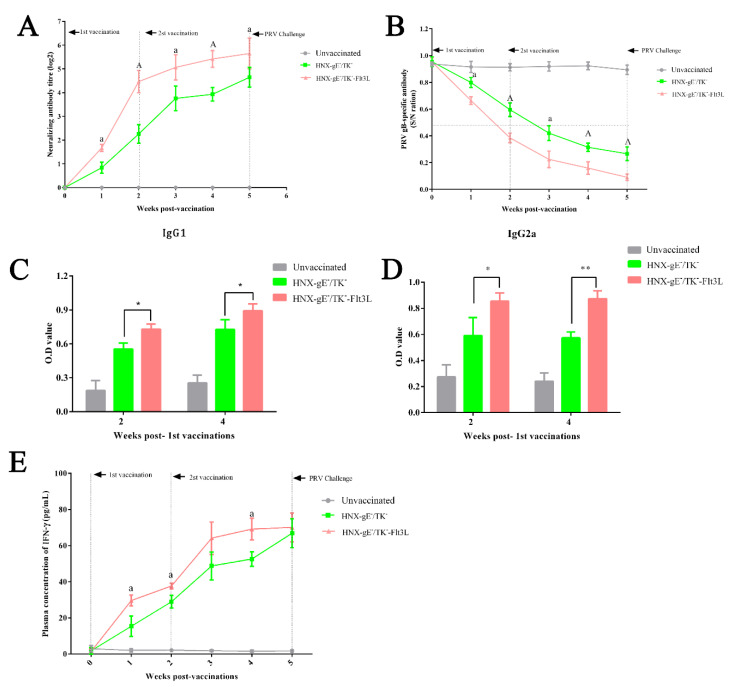
Immune responses after immunization with HNX-TK^−^/gE^−^-Flt3L in mice. Three groups of mice were intramuscularly injected with HNX-TK^−^/gE^−^, HNX-TK^−^/gE^−^-Flt3L or DMEM. (**A**) The neutralizing ability of antisera generated against HNX strain was calculated and expressed as the log. (**B**) gB-specific antibody ELISA. (**C**,**D**) The PRV-specific IgG1 and IgG2a titers were detected at two or four weeks post the first immunization through ELISA. (**E**) Cytokine IFN-γ ELISA. The data are the means from three independent experiments. *, *p* < 0.05; **, *p* < 0.01. a, *p* < 0.05; and A, *p* < 0.01. Note, the superscripts (a or A) in the picture indicated the differences between HNX-TK^−^/gE^−^-Flt3L vaccinated group with HNX-TK^−^/gE^−^ vaccinated group.

We further analyzed the immune responses elicited by HNX-TK^−^/gE^−^-Flt3L and HNX-TK^−^/gE^−^. First, a serum neutralization assay was performed to analyze the PRV antibody levels for different immunized groups. In the non-vaccinated group, no neutralizing antibody effective against PRV HNX strain infection was detected throughout the experiment. PRV-specific neutralizing antibodies of the HNX-TK^−^/gE^−^-Flt3L-vaccinated group and HNX-TK^−^/gE^−^-vaccinated group were detected at three-weeks post immunization and up to 5 weeks. At 5-weeks post vaccination, the neutralizing antibody titers of the HNX-TK^−^/gE^−^-Flt3L group (2^5.63^) were remarkably higher compared with the HNX-TK^−^/gE^−^ group (2^4.60^) (Figure 6A). Secondly, blocking ELISA analysis of serum samples was performed to examine PRV gB-specific antibody levels. A significantly stronger antibody response was found in HNX-TK^−^/gE^−^-Flt3L vaccinated group compared with in the HNX-TK^−^/gE^−^-vaccinated group at two weeks post-vaccination (Figure 6B). The gB-specific IgG antibodies isotypes (IgG1 and IgG2a) at 2 or 4 weeks post vaccination were further examined. As shown in Figure 6C,D, compared with the HNX-TK^−^/gE^−^-vaccinated group, the IgG1 and IgG2a levels were significantly elevated in HNX-TK^−^/gE^−^-Flt3L-vaccinated group. The concentrations of IFN-γ in the serum were tested using ELISA, and the results are shown in Figure 6E. As expected, the HNX-TK^−^/gE^−^-Flt3L-vaccinated group showed higher concentrations of IFN-γ, which might indicate a stronger immune response.

### 3.5. Protective Effects of HNX-TK^−^/gE^−^-Flt3L against Lethal PRV Infection

**Figure 7:**
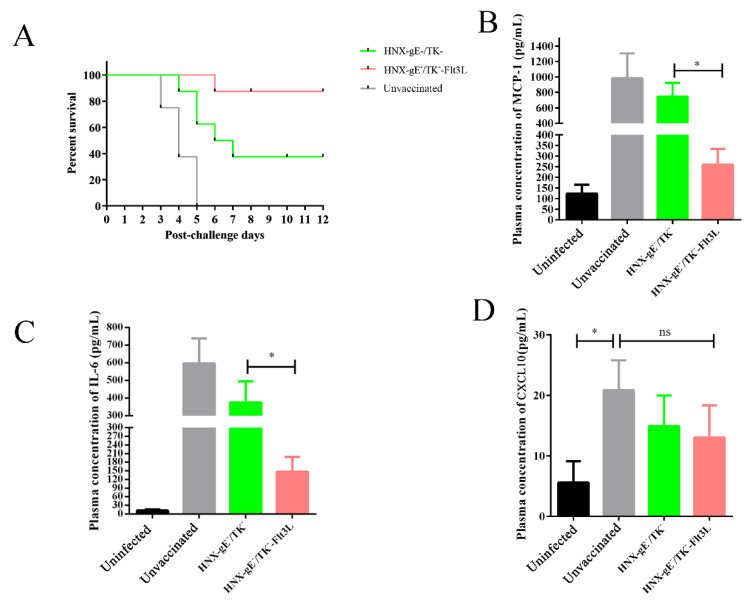
Protective effect of HNX-TK^−^/gE^−^-Flt3L in mice. Three groups of mice were intramuscularly immunised twice with HNX-TK^−^/gE^−^, HNX-TK^−^/gE^−^-Flt3L or DMEM, and then challenged with HNX strain. (**A**) Survival rate. (**B**) Cytokine MCP-1, (**C**) IL-6, and (**D**) CXCL10 levels in the plasma samples through cytokine ELISA at 72-h post challenge. *, *p* < 0.05.

To further investigate the protective effects of HNX-TK^−^/gE^−^-Flt3L, after three weeks of booster immunization, the immunized mice were challenged at 1 × 10^5^ TCID_50_ of wild type HNX strain by footpad injection. The survival and clinical symptoms of PR, e.g., pruritus, ruffled fur, and hyperkinesia, were observed for 2 weeks. In the HNX-TK^−^/gE^−^-Flt3L-vaccinated group, three mice showed clinical symptoms on day four post challenge. Two mice recovered over the following 2 days, and one mouse died (1/8). In the HNX-TK^−^/gE^−^-vaccinated group, seven mice showed clinical symptoms on day two post challenge, and five mice died (5/8). All mice in the control group died with severe pruritus (Figure 7A and Table 1). PRV infection induces a lethal systemic inflammatory response in mice [37]. Here, we performed ELISA assays to analyze the concentrations of inflammatory cytokines in the plasma three-days post challenge (Figure 7B–D). Compared to the non-vaccinated group, the lethal PRV infection induced much higher concentrations of IL-6, MCP-1, and CXCL10 in mice. In addition, compared to the HNX-TK^−^/gE^−^-vaccinated groups, HNX-TK^−^/gE^−^-Flt3L vaccination decreased the concentrations of IL-6 and MCP-1, but presented no significant changes in the level of CXCL10 at 72-h post challenge. 

**Figure 8:**
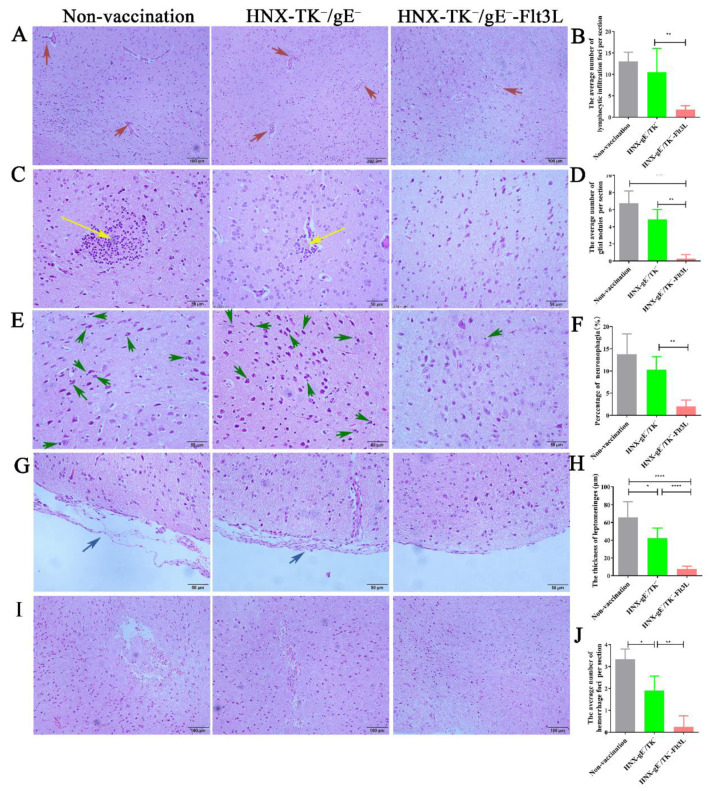
Pathological lesion of HNX-TK^−^/gE^−^-Flt3L and HNX-TK^−^/gE^−^ vaccinated mice. (**A**) Lymphocytic infiltration of mononuclear and lymphocytes cells near blood vessels (red arrow). (**B**) Quantification of lymphocytic infiltration foci. (**C**) Glial nodules and neurons (yellow arrow). (**D**) Quantification of glial nodules. (**E**) Neuronophagia (green arrow). (**F**) Quantification of neuronophagia. (**G**) Leptomeninges expansion (blue arrow). (**H**) Quantification of the thickness of leptomeninges. (**I**) Hemorrhage. (**J**) Quantification of hemorrhage foci. *, *p* < 0.05; **, *p* < 0.01 and **** *p* < 0.001.

Histopathological examination of brains was performed at 72 h post challenge. Sections, containing cerebrum and cerebellum, were subjected to histopathological examination. The most significant microscopic observation in non-vaccinated and HNX-gE^−^/TK^−^ -vaccinated group was mild nonsuppurative encephalitis in the cerebrum. In non-vaccinated and HNX-gE^−^/TK^−^-vaccinated group, we observed a slight lymphocytic infiltration of mononuclear and lymphocytes cells near blood vessels (Figure 8A,B), grey matter contained multifocal aggregates of glial cells (glial nodules) with affected neurons in center (Figure 8C,D), some affected neurons were surrounded by lymphocytes and glial cells (neuronophagia, Figure 8E,F). The leptomeninges expanded and contained small aggregates of lymphocytes, and increased clear space (edema, Figure 8G,H), and multifocal random accumulations of erythrocytes (hemorrhage, Figure 8I,J). The sections, obtained from HNX-gE^−^/TK^−^-Flt3L vaccinated group, had no significant alteration.

## 4. Discussion

The prevention of PR depends on the application of efficacious inactivated and live-attenuated vaccines [38]. An excellent PRV vaccine depends not only on a vaccine strain with good antigenicity but also on good adjuvants [39]. The commonly used adjuvants include mineral oil, chemicals such as aluminum hydroxide and aluminum hydroxide [40,41]. However, they are weak in the elicitation of cell-mediated immunity and are added into the vaccine during preparation, leading to complicated vaccine preparation procedures and high expenses [42]. Based on the immunomodulatory functions of Flt3L, in this study, we systematically evaluated the potential of Flt3L as an adjuvant in PRV vaccine development. A recombinant PRV with TK/gE gene-deletion and Ftl3L co-expression could provide both safety and immune adjuvant elements for one-step vaccine preparation.

The clinical effects of vaccines are influenced by several factors, including the adjuvants. Adjuvants can improve the immunogenicity and effective immunological memory through effects on antigen-presenting cells, such as DCs [39,43]. To improve the immunogenicity and simplify the vaccine preparation, the co-expression of some pleiotropic cytokines as molecular adjuvants have been attempted in PRV vaccine development; however, these attempts appear to have been beset with difficulties. Reportedly, Huihua Zheng et al. constructed a recombinant PRV expressing IL-6, but this did not stimulate a significantly higher level of PRV-specific neutralizing antibodies [44]. In our study, we constructed a recombinant TK/gE gene deleted PRV expressing Flt3L, as a key regulatory molecule in DC migration and activation. As expected, more DCs were activated by HNX-TK^−^/gE^−^-Flt3L both in vitro and in vivo. 

Choosing the right parental strain with good antigenicity is critical for vaccine efficacy [45]. Our previous studies investigated the growth characteristics and sequence analysis of the HNX strain. Compared with the Bartha K61 strain, the glycoprotein B and glycoprotein C genes of the HNX strain had 73 mutations [46]. Those mutations may the possible reason of immune failure. Here, we chose the HNX strain as the parental strain to construct HNX-TK^−^/gE^−^-Flt3L. Given the diversity of different PRV strains, we should investigate the cross-protection against different PRV emerging strains in future research [47].

Efficient vaccine production requires high viral titers, a prerequisite not always met by recombinant viruses. A previous study showed that a recombinant vaccinia virus that expressed Flt3L protein had a significantly lower virus titer in macrophage cells [48]. However, the expression of Flt3L by recombinant rabies did not affect the virus multiplication in BSR cells [49]. Here, we performed viral growth curve assays in PK-15 cells to assess the replication of HNX-TK^−^/gE^−^-Flt3L. In our study, similar replication kinetics were observed between HNX-TK^−^/gE^−^-Flt3L and the parental PRV HNX strain in PK-15 cells. The potential reason for this difference is that the influence of Flt3L on viral replication kinetics is virus and/or host cell specific. 

Systemic and lethal inflammatory responses are high during PRV infection in mice, and the signs of inflammation include heat, redness, oedema/swelling, pain, and loss of function of the injured area [37]. Even after vaccination, mice challenged with lethal PRV still showed high plasma levels of pro-inflammatory cytokines, such as IL-6 and MCP-1 (Figure 7 B–D). Inflammation caused by pathogens leads to undesirable consequences, potentially including organ and tissue damage and the unexpected diverting of nutrients away from productive purposes [50]. The HNX-TK^−^/gE^−^-Flt3L-vaccinated group challenged with lethal PRV had low plasma levels of IL-6 and MCP-1. One could speculate that the decrease of the inflammatory responses in lethal PRV infection may be beneficial to swine production. However, the alteration of cytokine formation/release remains to be clarified through mechanistic studies. 

A remarkable increase in neutralizing antibodies and gB-specific IgG antibodies were observed in mice with HNX-TK^−^/gE^−^-Flt3L vaccination. Higher levels of neutralizing antibodies and antigen-specific antibodies decreased the mortality rate, remitted the clinical signs, and mitigated the pathological damages. Despite the promising results, the improvement of IFN-γ was not sufficiently robust, and the clinical signs were not in complete remission. There are multiple studies that need to be performed, such as identifying appropriate vaccination dose and vaccination routes [51]. Finally, our animal experiments were performed in a mouse model, and HNX-TK^−^/gE^−^-Flt3L requires evaluation in pigs.

Our previous studies reported that the CRISPR/Cas9 and Cre/Lox systems are fast and cost-effective technologies for gene deletion vaccine development [25]. Here, we used those systems for the generation of recombinant vaccines that have foreign sequence insertions and virulence-associated gene deletions. In contrast with conventional recombination strategies and BAC clones, we can develop exogenous gene recombination and virulence-related gene deletion at the same time. The CRISPR/Cas9 system and Cre/Lox systems increased the multi-gene editing efficiency and decreased the time consumed during vaccine development.

## 5. Conclusions

The CRISPR/Cas9 and Cre/Lox systems were applied for the development of a TK/gE deleted and dendritic cell-targeted Flt3L expressing recombinant PRV vaccines candidate, HNX-TK^−^/gE^−^-Flt3L. The candidate exhibited a similar in vitro replication and growth kinetics to that of the parental PRV HNX strain. HNX-TK^−^/gE^−^-Flt3L enhanced DC recruitment and activation, induced robust PRV-neutralizing antibody and IFN-γ responses, decreased the mortality rate, clinical signs, and pathological damage following lethal PRV challenge. These results indicates that Flt3L is as promising adjuvant for a PRV vaccine and that PRV-HNX-TK^−^/gE^−^-Flt3L should be further evaluated as a safe and efficacious PRV vaccine.

## Figures and Tables

**Table 1:** The severity of clinical symptoms in mice of PRV challenge for 2 weeks.

Clinical Symptoms	Non Vaccinated	HNX-TK^−^/gE^−^	HNX-TK^−^/gE^−^-Flt3L
pruritus	8/8 + + +	2/8 +, 5/8 + +	1/8 + +
ruffled fur	8/8 + + +	7/8 + +	2/8 +, 1/8 + +
hyperkinesia	8/8 + + +	7/8 + +	3/8 + +

Severity of clinical symptoms: + mild symptoms; + + moderate symptoms; + + + severe symptoms.

## Data Availability

Data is contained within the article.

## Supplementary Materials

The following are available online at https://www.mdpi.com/article/10.3390/v13040691/s1, Table S1: Oligonucleotide primers used in this study, Figure S1: Sequence of PRV gE gene recombination donor template, Figure S2: Sequence of PRV TK gene recombination donor template.
