# Supplementary material for: Recombinant Pseudorabies Virus with TK/gE Gene Deletion and Flt3L Co-Expression Enhances the Innate and Adaptive Immune Response via Activating Dendritic Cells
Source: Viruses. 2021 Apr 16;13(4):691. doi: 10.3390/v13040691 (PMC8072707; doi:10.3390/v13040691)
Supplement: Supplementary file 1 [file viruses-13-00691-s001.zip › viruses-1105119-supplementary.pdf]

Supplementary Materials

Table S1. Oligonucleotide primers used in this study

Primers	Sequences	Application
sgRNA-TK-F	caccggtctgcgcatcgtaaatac	TK sgRNA cloning
sgRNA-TK-R	aaacgtatttacgatgcgcagacc	
sgRNA-gE-F	caccgccgtgttctttgtggcggt	gE sgRNA cloning
sgRNA-gE-R	aaacaccgccataaagaacacggc	
TKhm1-F	ccgccttatcatccccgctccccgccgccg	TKhm1 amplification
TKhm1-R	ggcgccgtcgaggtagatccggagg	
LoxP-TKhm2-F	ataacttcgtatagcatacattatacgaagttatgg tgtgaccctcgcccctcccacccgcg	TKhm2 amplification
TKhm2-R	ctccgcgtccgcgaggacggccac	
LoxP-CMV-F	ataacttcgtatagcatacattatacgaagttatgt gatgcggttttggcagtac	CMV enhancer amplification
CMV-R	agctctgcttatatagacc	
Flt3L-F	atggtagtgccggcgcca	Flt3L amplification
Flt3L-R	tcatgtgaatgctggggacac	
mCherry-F	atggtgagcaagggcgaggagga	mCherry amplification
mCherry-R	gggcccgggatccaccggatctaga	
gEhm1-F	gaacgggaccacggacctct	gEhm1 amplification
gEhm1-R	atctgcgcggcgcgcagcagaaagg	
loxN-eGFP-F	ataacttcgtatagtataccttatacgaagttatgtg agcaagggcgagg	eGFP amplification
eGFP- loxN-R	ataacttcgtataaggtatactatacgaagttatctt gtacagctcgtccat	
gEhm2-F	aaccgcctgttgatgtcc	gEhm2 amplification
gEhm2-R	acgcgctcgcgacaact	
gE300-F	cgggatctggacgttcctg	gE gene verification
gE500-R	atgggcggctcgcgccggag	
TK114-F	cgcactctgttcgacacgga	TK gene verification
TK367-R	gctgatgtccccgacgatga	
Flt3L 51-F	ctgctgctgctgctgctgag	Flt3L gene verification
Flt3L 392-R	gagatgttggcctggacgaagc	
eGFP-378-F	ggcatcgacttcaaggagg	eGFP gene verification
eGFP-665-R	tgatcccggcggcggtcacgaac	
mCherry-32-F	catcatcaaggagttcatgc	mCherry gene verification
mCherry-593-R	ccaacttgatgttgacgttg	

Fig. S1 Sequence of PRV gE gene recombination donor template (gEhm1-loxN-eGFP-loxN-gEhm2) gE hm1 and gE hm2 colored in green, loxP sites colored in grey, eGFP colored in blue, the endogenous TSS of PRV gE shaded in violet.

gaacgggaccacggacctctttgtgctgacggccctggtgccgccgagggggcgccccgtccccacgtcgccgcccgc
ggacgagtgccggcccgtcgtcggatcgtggcacgacagcctgcgcgtcgtggaccccgccgaggacgccgtgttcac
cacccagcccccgcccgagcccgagccgccgacgacccccgcgcccccccgggggaccggcgccacccccgagcc
ccgatcggacgaggaggaggagggtgacgcggagacgacgacgccgacgctgaccccggcgcccgggaccctgga
cgcgaacggcacgatggtgctgaacgccagcgtcgtgtcgcgcgtcctgctcgccgccgccaacgccacggcgggcg
cccggagccccgggaagatagccatggtgctggggcccacgatcgtcgtcctcctgatcttcctgggcgggatcgcctgc
gtggcccggcgctgcgcgcggaatcgcatctaccggccgcgacccgggcgcggatcggcggtccatgcggcgccccc
gcggcgcccgccccccaaccccgtcgccggggcgcccgtcccccagcccaagatgacgttggccgagctgcgccaga
agctcgccaccatcgcagaagaacaataaaaaggtggtgtttgcataattttgtgggtggcgttttatctccgtccgcgccgtt
ttaaacctgggcacccccgcgagtctcgcacacaccggggttgagaccatgcggccctttctgctgcgcgccgcgcagat
ataacttcgtatagtataccttatacgaagttatgtgagcaagggcgaggagctgttcaccggggtggtgcccatcctggtcg
agctggacggcgacgtaaacggccacaagttcagcgtgtccggcgagggcgagggcgatgccacctacggcaagctg
accctgaagttcatctgcaccaccggcaagctgcccgtgccctggcccaccctcgtgaccaccttcggctacggcctgca
gtgcttcgcccgctaccccgaccacatgaagcagcacgacttcttcaagtccgccatgcccgaaggctacgtccaggagc
gcaccatcttcttcaaggacgacggcaactacaagacccgcgccgaggtgaagttcgagggcgacaccctggtgaaccg
catcgagctgaagggcatcgacttcaaggaggacggcaacatcctggggcacaagctggagtacaactacaacagcca
caacgtctatatcatggccgacaagcagaagaacggcatcaaggtgaacttcaagatccgccacaacatcgaggacggc
agcgtgcagctcgccgaccactaccagcagaacacccccatcggcgacggccccgtgctgctgcccgacaaccactac
ctgagctaccagtccgccctgagcaaagaccccaacgagaagcgcgatcacatggtcctgctggagttcgtgaccgccg
ccgggatcactctcggcatggacgagctgtacaagataacttcgtatagtataccttatacgaagttataaccgcctgttgatg
tcccfgccccgcttaaataccgggagaaccggtccgcccgcattccgacatgcccggcgccgcctccgtcgacatggac
acgtttgaccccagcgcccccgtcccgacgagcgtctcgaacccggccgccgacgtcctgctggcccccaagggaccc
cgctccccgctgcgcccccaggacgactcggactgctactacagcgagagcgacaacgagacgcccagcgagttcctg
cgccgcgtgggacgccggcaggcggcgcgtcggagacgccgccgctgcctgatgggcgtcgcgatcagcgccgccg
cgctggtcatctgctcgctgtccgcgctactcgggggcatcgtcgccaggcacgtgtagcgagcgagcgagcgaacggg
agcgggggcccgcccccatccgccgcgcccaggagagggggagagagcgggggggttgggcgcgccacgtggtgt
gggcacggactcggacttgtcacaataaatgggccccggcgtgtccgggcgcacacagcagccttcctctcctccgcgtc
tctgttccgcccgtctctcgccggactcttcttctccaccgcctccaccgtcgcagttgtcgcgagcgcgt

Fig. S2 Sequence of PRV TK gene recombination donor template (TKhm1-Flt3L-loxP-CMV promoter-mCherry-loxP-TKhm2) TKhm1 and TKhm2 colored in green, Flt3L colored in yellow, loxN sites colored in grey, CMV enhancer colored in blue, and mCherry colored in red.

ccgccttatcatccccgctccccgccgccgcccggcccggccccgcgcgcgccgcgatcgcgatcaccgccgcggcc
cggcgacgtactcggcgaggccgcgcacggtcgcggccatcgcgctcgcgttgccgcgcgtctgggtgcagggcagg
cgcgtcacgtcgagcacgcgcatgctccgctgggccacaaacaccagcaggggcacgagcgtgatctcctcgccgccc
gggggcacggcggcggcgaggaggcgcgccgagtcgcgcagctggcacagcccctcgtgccgctgcccgcgcttgc
tgggcgtgttgaggttccgggggaagcggcacgtcttgagctcgatgaggaagcacaggtgcgggcccgcccccagcc
gcaccacgcacacgcagtcggggcggcgcaccccgaggttgacttcaaaggccagggtcaaggacgccttcttaagcg
tctctcggggaagcccgaagagactctcgccgtacgcggacgggtcgcgtcgcaggcgttcgtagaagcggttgtggca
gcggatccccgcccggaagcgcgccgggatgcgcatcctccggatctacctcgacggcgccatggtagtgccggcgcc
agcctggagcccaactacctcgctgctgctgctgctgctgctgctgctgctgagccccggccttctcgggagcccggactg
ctccttcccccacagccccatctcctccaccttcgccaacaccatccgccagctgtctgactacttgcttcaggattacccagt
cactgtcgcctccaacctgcaggacgacgagctctgcggggcgttctggcgcctggtcctggcccagcgttggatgggac
agctcaagaccgtggctgggtcccagatgcagaagctgctggaggctgtcaacaccgagatagtctttgtcacctcatgtg
ccctccagcccctccccagctgtcttcgcttcgtccaggccaacatctcccacctcctgcaggacacttcccagcagctggt
ggccttgaagccctggatcacccgccggaatttctcccgttgcctggagctgcagtgtcagccggacccctccaccctgct
gcccccaaggagtcctggggccttggaggccacatccctgccggcacctcaggcctctcttctgctcctcttgctcctgctg
ctgctgcctgccgccctcctgctgctggccactgcctggtgcctgcgccggaggaggaggagacggaggatgccctgcc
ccggggagcgggtgtccccagcattcacatgaataacttcgtatagcatacattatacgaagttatgtgatgcggttttggca
gtacatcaatgggcgtggatagcggtttgactcacggggatttccaagtctccaccccattgacgtcaatgggagtttgttttg
gcaccaaaatcaacgggactttccaaaatgtcgtaacaactccgccccattgacgcaaatgggcggtaggcgtgtacggt
gggaggtctatataagcagagctatggtgagcaagggcgaggaggataacatggccatcatcaaggagttcatgcgcttc
aaggtgcacatggagggctccgtgaacggccacgagttcgagatcgagggcgagggcgagggccgcccctacgagg
gcacccagaccgccaagctgaaggtgaccaagggtggccccctgcccttcgcctgggacatcctgtcccctcagttcatg
tacggctccaaggcctacgtgaagcaccccgccgacatccccgactacttgaagctgtccttccccgagggcttcaagtgg
gagcgcgtgatgaacttcgaggacggcggcgtggtgaccgtgacccaggactcctccctgcaggacggcgagttcatct
acaaggtgaagctgcgcggcaccaacttcccctccgacggccccgtaatgcagaagaaaaccatgggctgggaggcct
cctccgagcggatgtaccccgaggacggcgccctgaagggcgagatcaagcagaggctgaagctgaaggacggcgg
ccactacgacgctgaggtcaagaccacctacaaggccaagaagcccgtgcagctgcccggcgcctacaacgtcaacatc
aagttggacatcacctcccacaacgaggactacaccatcgtggaacagtacgaacgcgccgagggccgccactccacc
ggcggcatggacgagctgtacaagtccggactcagatctcgagctcaagcttcgaattctgcagtcgacggtaccgcggg
cccgggatccaccggatctagaataacttcgtatagcatacattatacgaagttatggtgtgaccctcgcccctcccacccgc
gccacggccggatggagaccgcgacggaggcaacgacgacggcgtgggagggggctcggggcgcgtataaagcca
tgtgtatgtcatcccaataaagtttgccgtgcccgtcaccatgcccgcgtcgtccgtgcgcctcccgctgcgcctcctgaccc
tcgcgggcctcctggccctcgcgggggccgccgccctcgcccgcggcgcgccgcagggtgggccgccctcgccgca
gggggtcccgcgcccaccgcggcgcccgcgcgcgggcccaccctgttcgtcctggacggcgacggctccgcgtggt
tcgtcttccagctcggcgggctgggggcgctcaacgacacgcgcatccgcgggcacctgctcggccggtacctcgtctc
gtaccaggtggtgcccccgcccgtctccgcgtggtactttgtgcagcgcccgcgcgagcgcccgcgcctctcggggccg
ccctcgggcgcggagctcgtggccttcgacgcgcccggcgtccggcgcacgtacaccacggcggcggtgtggcccgc
ggaggtggccgtcctcgcggacgcggag
